# Knowledge, attitudes, and behaviours about gender equality: a cross-sectional survey of adolescents from rural India

**DOI:** 10.12688/f1000research.124577.1

**Published:** 2022-09-13

**Authors:** Anand Ahankari, Pavel Ovseiko, Pratyush Kabra, Sneha Giridhari, Kranti Rayamane, Clare Whitfield, Parveen Ali, Mark Hayter

**Affiliations:** 1 Faculty of Health Sciences, University of Hull, Hull, HU6 7RX, UK; 2 Faculty of Health & Medical Sciences, University of Surrey, Guildford, GU2 7YH, UK; 3 Radcliffe Department of Medicine, University of Oxford, Oxford, OX3 9DU, UK; 4 Department of Community Medicine, Ashwini Rural Medical College, Hospital and Research Centre, Solapur, Maharashtra, 413006, India; 5 SWISSAID, Pune, Maharashtra, 411040, India; 6 Halo Medical Foundation, Andur, Maharashtra, 413603, India; 7 School of Nursing and Midwifery, University of Sheffield, Sheffield, S10 2LA, UK; 8 Faculty of Health, Psychology and Social Care, Manchester Metropolitan University, Manchester, M15 6BH, UK

**Keywords:** Gender equality, Adolescent, Sustainable Development Goals, Gender-based discrimination, Violence, Domestic violence, India, Maharashtra.

## Abstract

**Background:** Gender equality is a fundamental human right and vital to accelerate global progress towards several Sustainable Development Goals (SDGs). Adolescents’ involvement is essential to achieve such equality and SDGs to develop peaceful sustainable societies. However, there are limited data especially from developing countries such as India to plan gender equality related programmes targeted at adolescents.

**Methods**: We conducted a cross-sectional survey to assess gender equality related knowledge, attitudes, and behaviours among 16 to 19 year-old adolescents from sixty villages of the Maharashtra state of India.

**Results**: Data from 1306 respondents (667 females and 639 males) showed a mean score of 30 out of 44, suggesting an overall moderate gender equality score in rural adolescents. The majority of girls (68.3%) were in the high scoring group, whereas the majority of boys were in the moderate group (60.3%). Regression analysis showed that responses from boys were associated with lower scores compared to responses from girls by five points (adjusted β-coefficient: -4.99, 95%CI: -5.85 to -4.12, p<0.001).

**Conclusions**: Our findings suggest that there is a need to involve adolescents with a major focus on boys to improve gender equality in rural areas of Maharashtra. This will help introduce concepts of equality from an early age to educate boys, empower girls, and address gender-based discrimination and violence against girls and women.

## Introduction

Achieving gender equality and empowering all women and girls is the fifth Sustainable Development Goal (SDG). The SDGs were adopted by all United Nations Member States in 2015 ( United Nations, 2021). Gender equality is also vital for other SDGs such as ending poverty; ensuring inclusive and equitable quality education; fostering sustained, inclusive, and sustainable economic growth; and reducing inequality. India, home to one-sixth of the world’s population, has experienced enormous economic and technological developments since the globalisation. Although India has an important role to play in accelerating global progress towards gender equality, improvement in gender equality has been slow. Gender-based discrimination and inequalities in India are widely documented ( Sumanjeet, 2016) making it one of the most challenging countries in the world to be female ( Landry *et al*., 2020). The 2019 gender inequality index reports India at 123 ^rd^ position (out of 162 nations), suggesting a range of challenges faced by Indian females ( United Nations, 2022).

Gender-based discrimination creates an oppressive environment for young girls and women. It is deeply rooted in the country’s culture, society, and practices. One example of this is sex selective abortions (pregnancy termination on identifying female child due to preference to have a male offspring) that have disturbed the natural sex ratio in India ( Ahankari *et al*., 2015a, National Institution for Transforming India (NITI), 2015). Gender-based discrimination manifests from an early age and continues over the life course, e.g. neglect of a female child from birth, poor nutrition provision, lack of education support and child marriages ( Brahmapurkar 2017, Davis *et al*., 2013, UNICEF 2017). Girls often have limited school progression opportunities and high dropout rates compared to boys. Gender discrimination and related practices/behaviours often form part of community and societal culture, and become social and cultural norms ( Sumanjeet 2016, Jayachandran 2020, Mukhopadhyay *et al*., 2019). Such norms are likely to impact on the health of women from early childhood into adulthood, as well as on their family health. Studies report higher mortality in female babies during the postnatal period and also during early childhood compared to boys ( Patra 2011, Bobbitt-Zeher 2007). Furthermore, child marriages disproportionately increase the health and emotional burden of early pregnancy and also increase chances of maternal mortality in young females ( Fan and Koski, 2022). Lower socioeconomic status, lack of employment/formal earning source, and absence of active role in decision making processes within the family, may disadvantage women in accessing health services, for personal and their children’s needs, especially for a female child ( UN Secretary General’s High Level Panel on Women’s Economic Empowerment, 2022). Therefore, gender inequality substantially affects women’s quality of life over time, and places them at a higher risk of physical, emotional, and sexual exploitation ( Brahmapurkar 2017, Davis *et al*., 2013, UNICEF 2017, Jayachandran 2020, Mukhopadhyay *et al*., 2019). Oppressive gender norms also contribute to violence against women in public and private spheres, and this may explain the fact that a large number of Indian women (30-50%) face intimate partner violence ( Kalokhe *et al*., 2016, Krishnamoorthy *et al*., 2020). However, prevalence of violence could be much higher because it is often under-reported due to poor governance, victim’s fear, a lack of essential support services, and societal views towards such abuse ( Kalokhe *et al*., 2016, Krishnamoorthy *et al*., 2020). Economic impacts of gender inequality are also high, affecting India’s gross domestic product and limiting national economic development ( Jayachandran 2020, Arora 2012).

The Indian government, non-government organisations, and national and international aid agencies are implementing a range of programmes to address gender inequalities and reduce/prevent violence against women and girls. Recently, such programmes have seen a growing focus on the involvement of men, with an aim to increase awareness of gender equality among them, reduce violence against girls/women, improve family relations and address sustainability of such family and community level interventions ( SWISSAID 2021, Ahankari *et al*., 2015b). Along with the interventions in the adult population, education and training aimed at adolescents are essential for the prevention of gender-based violence and discrimination in future generations. Projects aimed at developing evidence based strategies for adolescents and young men are gaining importance ( Freudberg *et al*., 2018, Jejeebhoy *et al*., 2017). However, there is limited published research on gender equality about knowledge, attitudes, and behaviours in Indian adolescents especially from rural areas of the country. About 20% of India’s total population consists of adolescents (10-19 years), and approximately 70% of these are in rural areas ( Census of India, 2021), often in difficult to access communities. The majority of the existing research on gender equality has focussed on urban settings. There are no published data from the Maharashtra state of India involving adolescents on their outlined gender parameters. Our study is designed to address this gap in the literature by assessing gender equality related knowledge, attitude, and behaviours in adolescent boys and girls in a rural area of Osmanabad district; specifically, one of the difficult to access areas of the Maharashtra state of India. Our study will also provide vital information to plan future development work, where adolescents could be involved in gender equality initiatives/projects.

## Methods

### Ethics

The study has been reviewed and approved by the Faculty of Health Sciences Ethics Committee, University of Hull, UK (Reference number FHS125), and also by the ethics committee of the Ashwini Rural Medical College, Hospital and Research Centre, Solapur, Maharashtra, India (Reference number ARMCH/IECHR/03/2019). Verbal informed consent was obtained from all participants, and participants were asked to report at a data collection site only if they wished to participate in the study.

### Study aims and objectives

This quantitative study was conducted as part of the DEVELOP project initiative ( Ahankari *et al*., 2021). The DEVELOP (phase 1) project had two components: a quantitative survey of adolescents and a qualitative evaluation of the *Responsible Couples* project. This paper reports findings from the survey that assessed knowledge, attitudes, and behaviours (KAB) about gender equality in 16 to 19-year-old adolescent boys and girls from rural areas of the Maharashtra state of India (quantitative component of the DEVELOP phase 1 project). The key objectives of the quantitative component were to develop and use a survey instrument, to analyse data collected through the survey and to test the reliability of the survey instrument.

### Study setting

The study was conducted in 60 villages from the Osmanabad district of the Maharashtra state of India. Our local project partner, the Halo Medical Foundation (HMF), has been working in this area since 1993 through health and development programmes including the *Responsible Couples* project, funded by the SWISSAID, Switzerland (international aid agency) to address gender-based violence against women. However, no gender equality interventions specifically targeted at adolescents have been implemented in this area prior to our research. The study area was primarily selected considering access to population and future project strategy, where our local collaborators (HMF and SWISSAID) can support field based research activities. The nearest village is approximately 3 kilometres from the HMF/research centre, and the farthest is up to 60 kilometres. Travel infrastructure including road and transport are limited in this rural region. Osmanabad is one of the marginalised districts in the Maharashtra state and also nationally in India, however, in the recent decade the area has experienced some social and economic developments ( Yashada, 2012). Despite this, the human development index of the district (0.649) as well as the literacy rate, remains low ( Yashada, 2012). Considering findings in Yashada’s 2012 report, approximately half of the population live in good dwelling conditions (50-60%), while more developed regions of the Maharashtra state have a higher proportion with better living conditions (over 70% in areas such as Mumbai, Pune, Kolhapur districts) ( Yashada, 2012). For our study area, additional indicators, such as total literacy rate (76.33%) and per capita income, also remain low compared to the majority of the districts across the state ( Yashada, 2012). The 2011 literacy data showed a higher proportion than the national literacy rate for the district, but lower than the state’s average and other areas of Maharashtra ( Yashada, 2012). Literacy rates in women are lower (66.67%) than men (85.31%) and is below the state’s average (75.48% literacy among women in Maharashtra) ( Yashada, 2012). There are no published data on gender equality related indicators specific to our region.

### Study population

Girls and boys aged 16 to 19 years old living in the study area (the 60 villages of Osmanabad district), who were able to read and write in the local language, Marathi, were eligible to participate in the study. The study protocol and research procedures were peer reviewed and published ( Ahankari *et al*., 2021). These are briefly summarised below.

### Study design

The survey instrument was developed by adapting the Gender Equitable Measurement (GEM) Scale from previous research in an urban setting in India, to the given rural population ( Achyut *et al*., 2011). We first reviewed nine previously published gender equality scales ( Achyut *et al*., 2011, Nanda 2011), and identified the GEM scale with fifteen items as the most relevant to our study aim and scope. We then adapted it to the given rural setting and research needs through discussions with our project partners, research team members, project staff, and local stakeholders. Namely, we reworded thirteen items to improve clarity, provided examples for two items, excluded two items to avoid duplication, and added nine new items. We then translated the survey instrument into Marathi language and checked its face validity through discussions involving over fifty adolescents from our study area. Following these discussions, we amended two questions to improve its clarity and relevance to the given study setting. The final survey instrument had twenty-two statements; nine statements on knowledge related to gender beliefs and gender-based role allocation, six statements on attitudes towards education resources and environment, and seven statements on behaviour related to violence (see *Extended data* ( Ahankari *et al*., 2019)). The survey instrument also had nine questions on demographics and household resources, which were derived from two previous research projects conducted in the same geographic region ( Ahankari *et al*., 2017a, 2017b).

### Data collection

**Figure 1.**
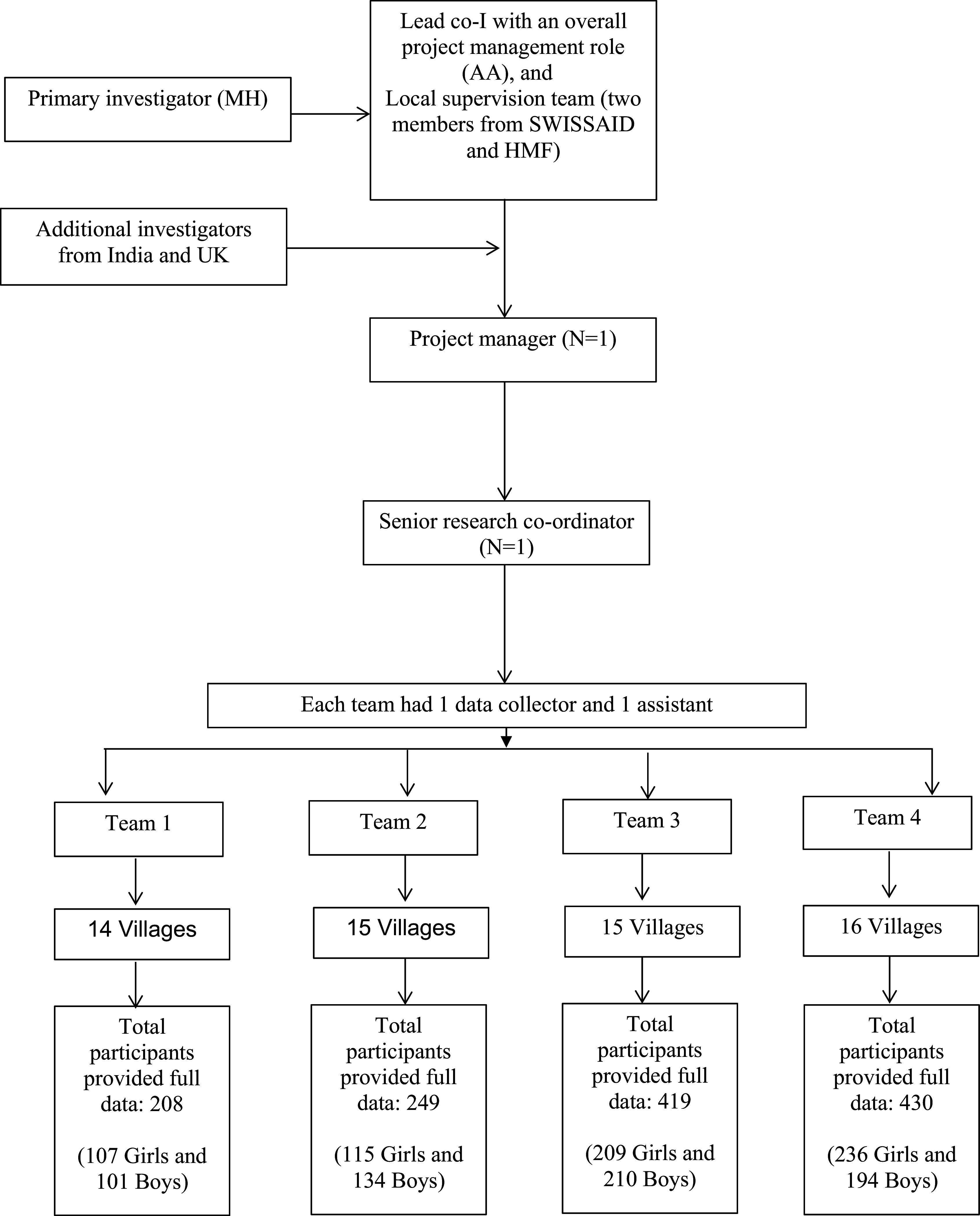
Project management structure and survey recruitment.

Four data collection teams under supervision of a senior research co-ordinator (Ms Sandhya Rankhamb) and a project manager (Ms Shilpa Toro) collected the data between April and July 2019 ( Figure 1, details on field staff contributions are available in the acknowledgments). Written information about the survey (PIS- Participant Information Sheet) was provided in each village to eligible participants at least two weeks in advance (see *Extended data* ( Ahankari *et al*., 2019)). The PIS included details about the project background, data collection processes, consent, type of data, and its future application including intention to publish research outputs from this work. These were also verbally explained by field based data collection staff (details on field staff contributions are available in the acknowledgments). Details of data collection day and exact location in each village were informed verbally by members of the data collection team to eligible participants. Two weeks were offered to eligible participants to make an informed decision on whether or not they wished to participate in the study. Due to the nature of the research, subject area, and ethics permissions, no personal details such as name or address, were collected at any time across the project duration from participants. On the day of the data collection, informed verbal consent was explained to participants and those who were willing to participate continued to the next stage. Each participant was then given a survey form prepared in the local language (Marathi) along with an envelope to submit when completed. For those who had questions prior to their participation, these were answered verbally by members of the project team at the data collection site. The data collection sites included a range of location across study villages such as but not limited to schools, health centres, village community halls, or community spaces depending on availability and suitability to conduct village based data collection activities. Once data forms are completed, participants dropped those in a sealed envelope containing the form in a secure box made available at the site. Participants took approximately 30 minutes to complete and return the questionnaire. On submitting a completed form in a sealed envelope, participants received a textbook to take home (reading material in Marathi) on gender equality, thanking them for their contribution to this study. The same reading material was provided to each study participant, and cost of each textbook was 40 Indian rupees (approximately £0.50), which was covered through project funds. Participation was voluntary and there were no monetary benefits to survey respondents. All field based data collection activities were completed between April and July 2019.

### Data quality and research conduct

All project staff received training across the research duration and were closely monitored on a daily and weekly basis through a systematic approach. For example, induction training was conducted in the local language by one of the lead researchers (AA) followed by capacity building exercises to ensure research and ethics conduct. Four teams were created, each having one female healthcare worker and one graduate staff with social and development work experience. This was supervised by a senior research co-ordinator, who had over five years of research experience with the lead researcher (AA), gained through implementation of three UK-India research projects conducted prior to the DEVELOP phase 1 work. The senior research co-ordinator monitored all four data collection teams on a daily basis across the project duration, and was also supervised by a project manager on a weekly basis. Additional research trainings were conducted prior to the start of data collection session and weekly staff meetings were facilitated by a project manager. Study progress reporting system was used to share details with all India and UK collaborators on a monthly basis, and it was prepared and signed off by senior research co-ordinator and project manager. During field based data collection, visits and inspections were undertaken by both the senior research co-ordinator and manager, to ensure quality and conduct was in line with study procedures. All data collected on paper based forms were entered into Microsoft Office Excel, and were checked twice independently by two members of field staff (data collector and senior research co-ordinator), and was signed off by an on-site project manager in India (details on field staff contributions are available in the acknowledgments). One of the co-investigators (PK) conducted inspection visits from an ethics perspective and also provided inputs to improve processes such as steps on how to check completed data forms, writing progress reports, and data storage plan. Data cleaning and management steps were discussed and explained to all staff and collaborators to ensure transparency, and feedback was obtained to improve systems. Lead author and researcher (AA) implemented research projects ( Ahankari *et al*., 2017a, 2017b) in the capacity of a primary investigator prior to the DEVELOP phase 1 work in the same geographic region in India, and used this experience to design required data collection and monitoring process outlined above to ensure study quality and research conduct.

### Analysis

The primary outcome of interest was the total KAB score. Each statement was scored on a three-point scale from 0 to 2 [0 - agree, 1 - partially agree, 2 - disagree] ( Achyut *et al*., 2011). Responses to all statements were summed into the total KAB score, which could range from 0 to 44. The total KAB score was used as a continuous outcome for the linear regression analysis. Furthermore, the KAB score was trichotomised into low [0-15], moderate [16-30], and high score groups [31-44] ( Achyut *et al*., 2011). Demographics and household resources (independent) data variables collected on individuals were used to investigate their associations with the outcome of interest (KAB score). All independent variables were included in the fully adjusted linear regression. The significance is determined based on a p value < 0.05. Cronbach’s alpha internal consistency coefficients were estimated to assess the reliability of the survey instrument/scale ( Boroumandfar *et al*., 2016, Shi *et al*., 2014). Stata 13.1 was used for the statistical analyses (StataCorp, College Station, Texas, USA). The study is reported in line with the STROBE guidelines ( von Elm *et al*., 2007).

## Results

In total, 1445 adolescents from 60 villages completed the self-reported questionnaire (data forms, of which 139 questionnaires were removed prior to the analysis during quality checks (N=75 boys, N=64 girls). Primary reason for elimination were as follows; multiple answers reported for one or several question(s), re-written or modified answer(s), and incomplete data forms where one or more questions were left unanswered. This elimination was a part of the data quality work which was described in detail in the methods having a systematic process (data entry, error identification, cleaning, quality check and approval by senior staff). All eliminated forms (N=139) were checked by a senior research co-ordinator and project manager who agreed on this elimination, and details of the same were presented to all staff and key collaborators. Complete data from 1306 participants were used in the analysis. Of these, 51% were girls (N=667), and 49% were boys (N=639). The study recorded a high response rate across field area (90% of the distributed forms were returned with full data). About 62% of the total participants were 16 to 17 years old, and the lowest proportion were 19 years old ( Table 1). The majority of the adolescents (93%) were in a full-time education (attending school or a college), and about 20% were engaged in a formal/informal paid employment ( Table 1).

**Table 1.** Characteristics of study participants with linear regression analysis (N=1306).

Characteristics	Frequency (percentage)		Unadjusted analysis	Fully adjusted analysis
*Age*				
16	484 (37.06%)		Reference	Reference
17	327 (25.04%)		-0.88 (-1.96 to 0.19)	0.06 (-0.94 to 1.06)
18	274 (20.98%)		-0.69 (-1.83 to 0.44)	0.23 (-0.84 to 1.32)
19	221 (16.92%)		-0.66 (-1.88 to 0.56)	0.85 (-0.34 to 2.05)
*Age distribution by sex*				
	Girls	Boys	NA	NA
16	276 (41.37%)	208 (32.55%)		
17	150 (22.48%)	177 (27.69%)		
18	140 (20.98%)	134 (20.97%)		
19	101 (15.14%)	120 (18.77%)		
*Sex*				
Girls	667 (51.07%)		Reference	Reference
Boys	639 (48.93%)		-5.87 (-6.64 to -5.10) **	-4.99 (-5.85 to -4.12) **
*Village location*				
Lohara block	849 (65.01%)		Reference	Reference
Tuljapur block	457 (34.99%)		0.09 (-0.78 to 0.96)	0.35 (-0.47 to 1.17)
*Attends school/college*				
No	85 (6.51%)		Reference	Reference
Yes	1,221 (93.49%)		0.45 (-1.23 to 2.14)	0.79 (-0.79 to 2.39)
*Paid job*				
No	1,042 (79.79%)		Reference	Reference
Yes	264 (20.21%)		-4.46 (-5.47 to -3.45) **	-2.57 (-3.59 to -1.54) **
*House structure*				
Permanent	313 (23.97%)		Reference	Reference
Semi-Permanent	931 (71.29%)		-0.10 (-1.09 to 0.87)	0.13 (-0.79 to 1.07)
Temporary	62 (4.75%)		-1.54 (-3.63 to 0.55)	0.03 (-1.95 to 2.01)
*Parents own a television set*				
No	360 (27.57%)		Reference	Reference
Yes	946 (72.43%)		1.23 (0.30 to 2.17) *	0.72 (-0.17 to 1.61)
*Parents own a mobile phone*				
No	115 (8.81%)		Reference	Reference
Yes	1,191 (91.19%)		1.64 (0.17 to 3.11) *	-0.53 (-1.97 to 0.89)
*Personal mobile phone*				
No	878 (67.23%)		Reference	Reference
Yes	428 (32.77%)		-3.46 (-4.33 to -2.59) **	-0.99 (-1.94 to -0.05) *

Linear regression reported using β coefficient with 95% confidence intervals. Total KAB score was calculated using the DEVELOP scale, which was the primary outcome of interest for the regression analysis. Minimum score was 0 and maximum was 44, and the total KAB score of each participant was used as a continuous outcome in the linear regression model. All independent variables (reported in the table) were used in the fully adjusted analysis.

NA - Not applicable.

* Indicates p value <0.05.

** indicates p value <0.001.

Reponses pertaining to the KAB scale are detailed in Table 2. The mean gender equality score was 30 [Standard deviation 7.68, Range 0 to 44], suggesting an overall moderate gender equality (GE) scores in rural adolescents in our study area. Less than 4% were in the low GE group (score- 0 to 15), 45% were in the moderate (score- 16 to 30), and over 50% were in the high GE group (score- 31 to 44). The majority of girls (68%) were in the high scoring group, whereas the majority of boys were in the moderate group (60%). Regression analysis reported that responses from boys were associated with lower scores compared to girls by 5 points, when assessed using our KAB scale (adjusted β coefficient -4.99, 95% CI -5.85 to -4.12, p<0.001) ( Table 1). Those who were in formal/informal paid employment were also associated with lower scores by 2 to 3 points (adjusted β coefficient -2.57, 95% CI -3.59 to -1.54, p<0.001). Those with a personal mobile phone were likely to have a low score but such effect was minimal (adjusted β coefficient -0.99, 95% CI -1.94 to -0.05, p<0.05). Only 33% of our study participants owned a personal mobile phone and the majority of them were boys. Cronbach’s alpha internal consistency coefficient for the KAB scale was 0.82 indicating sufficiently high reliability of the scale.

**Table 2.** DEVELOP gender equality scale (N=1306).

Scale component and questions in each component	Participant’s response	Frequency (%) Includes both boys and girls (N=1306)	Response by girls (%), total girls (N=667)	Response by boys (%), total boys (N=639)
Knowledge related to gender beliefs and gender-based role allocation (9 questions)				
1. Only men or boys should work outside the house. Example: on agricultural farm or other paid jobs.	Agree	251 (19.22%)	71 (10.64%)	180 (28.17%)
	Partially agree	177 (13.55%)	72 (10.79%)	105 (16.43%)
	Disagree	878 (67.23%)	524 (78.56%)	354 (55.40%)
2. Men need more care and support as they work harder than women.	Agree	622 (47.63%)	256 (38.38%)	366 (57.28%)
	Partially agree	388 (29.71%)	232 (34.78%)	156 (24.41%)
	Disagree	296 (22.66%)	179 (26.84%)	117 (18.31%)
3. The husband or father or brother (male person in the family) should decide to buy any major household items.	Agree	240 (18.38%)	89 (13.34%)	151 (23.63%)
	Partially agree	220 (16.85%)	95 (14.24%)	125 (19.56%)
	Disagree	846 (64.78%)	483 (72.41%)	363 (56.81%)
4. A man or brother or father (male person in the family) should have the final word about decisions at home.	Agree	294 (22.51%)	121 (18.14%)	173 (27.07%)
	Partially agree	269 (20.60%)	128 (19.19%)	141 (22.07%)
	Disagree	743 (56.89%)	418 (62.67%)	325 (50.86%)
5. A woman’s role is taking care of her house and family members. Example: Childcare, cooking and looking after elderly people or other family members.	Agree	636 (48.70%)	303 (45.43%)	333 (52.11%)
	Partially agree	346 (26.49%)	175 (26.24%)	171 (26.76%)
	Disagree	324 (24.81%)	189 (28.34%)	135 (21.13%)
6. A woman should obey her husband in all matters and scenarios.	Agree	437 (33.46%)	139 (20.84%)	298 (46.64%)
	Partially agree	362 (27.72%)	213 (31.93%)	149 (23.32%)
	Disagree	507 (38.82%)	315 (47.23%)	192 (30.05%)
7. A good woman never questions her husband’s opinions, even if she is not sure she agrees with them.	Agree	434 (33.23%)	194 (29.09%)	240 (37.56%)
	Partially agree	403 (30.86%)	222 (33.28%)	181 (28.33%)
	Disagree	469 (35.91%)	251 (37.63%)	218 (34.12%)
8. It is necessary to give dowry or other offerings at the time of marriage of a girl.	Agree	193 (14.78%)	72 (10.79%)	121 (18.94%)
	Partially agree	193 (14.78%)	79 (11.84%)	114 (17.84%)
	Disagree	920 (70.44%)	516 (77.36%)	404 (63.22%)
9. The only thing parents can really rely on in their old age is their sons.	Agree	502 (38.44%)	200 (29.99%)	302 (47.26%)
	Partially agree	356 (27.26%)	199 (29.84%)	157 (24.57%)
	Disagree	448 (34.30%)	268 (40.18%)	180 (28.17%)
Attitudes towards education resources and environment (6 questions)				
10. Girls cannot do well at school and overall in their life compared to boys.	Agree	217 (16.62%)	91 (13.64%)	126 (19.72%)
	Partially agree	173 (13.25%)	69 (10.34%)	104 (16.28%)
	Disagree	916 (70.14%)	507 (76.01%)	409 (64.01%)
11. Boys are naturally better at studies and perform better at school compared to girls.	Agree	183 (14.01%)	45 (6.75%)	138 (21.60%)
	Partially agree	253 (19.37%)	91 (13.64%)	162 (25.35%)
	Disagree	870 (66.62%)	531 (79.61%)	339 (53.05%)
12. Boys are naturally better than girls in all types of sports and activities.	Agree	274 (20.98%)	62 (9.30%)	212 (33.18%)
	Partially agree	285 (21.82%)	124 (18.59%)	161 (25.20%)
	Disagree	747 (57.20%)	481 (72.11%)	266 (41.63%)
13. If there is a limited amount of money at home to pay for education, it should be spent on sons first.	Agree	440 (33.69%)	185 (27.74%)	255 (39.91%)
	Partially agree	369 (28.25%)	197 (29.54%)	172 (26.92%)
	Disagree	497 (38.06%)	285 (42.73%)	212 (33.18%)
14. It is important that boys have more education than girls.	Agree	219 (16.77%)	70 (10.49%)	149 (23.32%)
	Partially agree	232 (17.76%)	69 (10.34%)	163 (25.51%)
	Disagree	855 (65.47%)	528 (79.16%)	327 (51.17%)
15. Since girls have to get married, they should not be sent for schools or higher education.	Agree	83 (6.36%)	30 (4.50%)	53 (8.29%)
	Partially agree	108 (8.27%)	36 (5.40%)	72 (11.27%)
	Disagree	1115 (85.38%)	601 (90.10%)	514 (80.44%)
Behaviour related to violence (7 questions)				
16. Girls like to be teased by boys.	Agree	71 (5.44%)	12 (1.80%)	59 (9.23%)
	Partially agree	117 (8.96%)	27 (4.05%)	90 (14.08%)
	Disagree	1118 (85.60%)	628 (94.15%)	490 (76.68%)
17. Girls provoke boys with short or fancy dresses.	Agree	246 (18.84%)	59 (8.85%)	187 (29.26%)
	Partially agree	269 (20.60%)	114 (17.09%)	155 (24.26%)
	Disagree	791 (60.57%)	494 (74.06%)	297 (46.48%)
18. It is a girl’s fault if a boy or a male teacher harasses her in any form.	Agree	145 (11.10%)	46 (6.90%)	99 (15.49%)
	Partially agree	206 (15.77%)	93 (13.94%)	113 (17.68%)
	Disagree	955 (73.12%)	528 (79.16%)	427 (66.82%)
19. A woman or a girl should tolerate violence at home in order to keep her family together.	Agree	134 (10.26%)	47 (7.05%)	87 (13.62%)
	Partially agree	203 (15.54%)	79 (11.84%)	124 (19.41%)
	Disagree	969 (74.20%)	541 (81.11%)	428 (66.98%)
20. There are times when a woman or a girl deserves to be beaten by her husband or father or brother.	Agree	143 (10.95%)	57 (8.55%)	86 (13.46%)
	Partially agree	262 (20.06%)	126 (18.89%)	136 (21.28%)
	Disagree	901 (68.99%)	484 (72.56%)	417 (65.26%)
21. A man using violence against his wife, or a brother using violence against his sister or mother is a private matter that shouldn’t be discussed outside the house.	Agree	298 (22.82%)	112 (16.79%)	186 (29.11%)
	Partially agree	283 (21.67%)	160 (23.99%)	123 (19.25%)
	Disagree	725 (55.51%)	395 (59.22%)	330 (51.64%)
22. If a girl rejects a boy, he should defend his reputation with force including physical if he has to.	Agree	109 (8.35%)	38 (5.70%)	71 (11.11%)
	Partially agree	163 (12.48%)	68 (10.19%)	95 (14.87%)
	Disagree	1034 (79.17%)	561 (84.11%)	473 (74.02%)

## Discussion

To the best of our knowledge, this is the first study measuring GE related knowledge, attitudes, and behaviours among adolescents in rural and difficult to access areas of the Maharashtra state of India, where information on gender equality was limited. Although the Gender Equitable Measurement (GEM) Scale and its modifications have been previously applied in urban population in India, GE related knowledge, attitudes, and behaviours among rural adolescents in Maharashtra and also broadly in India were limited. A sufficiently high Cronbach’s alpha coefficient of our survey instrument indicates that our scale offers reliable measurement of gender equality in rural Indian setting.

Our findings demonstrate a statistically significant difference in GE related knowledge, attitudes and behaviours between boys and girls. Adolescent boys scored significantly lower than girls when assessed using our scale, suggesting needs to develop projects to include boys/young men from an adolescent/early stage through education and training initiatives. Our research findings are in agreement with studies conducted elsewhere in India ( Syed 2017, Landry *et al*., 2020). A study from Hyderabad city of India involving 186 adolescents (13 to 16 years) showed similar results ( Syed 2017). Girls scored higher than boys in both pre and post intervention assessments. Pre-intervention average score in boys was 21.51 (out of 36), while in girls it was 24.8. Post-intervention gender score difference was significant where girls again scored higher (34.08 out of 36) than boys (24.63 out of 36). Cross-sectional data from 1,691 participants (8 to 18 years) from three Northern Indian states namely Delhi, Punjab, and Rajasthan) showed that boys scored lower compared to girls when assessed for gender-equitable attitudes ( Landry *et al*., 2020). The scores were analysed similar to our study (low, medium, and high categories), but were further assessed by school year/grade and also by geographic region. Findings showed that the overall GE related attitudes improved with age/grade, but boys were likely to be in the lowest scoring categories and girls scored higher over time, which is similar to our research findings. Das and associates examined gender attitudes and violence among urban boys in Mumbai, India ( Das *et al*., 2014). The survey involved 1040 boys aged 10 to 16 years, who were recruited as a part of a larger intervention project. Data on gender related attitudes were collected using a scale similar to our survey tool. The study reported high prevalence of inequitable gender attitudes condoning violence against girls. Only one in five study participants (from 10-12 years age group) reported high levels of condoning violence where the rest suggested some acceptance of such behaviours considering their personal and community exposures/experiences. Those with moderate and equitable attitudes were less likely to engage in violence against girls/women. Such associations remained significant on further subgroup analyses where boys with and without a history of personal violence exposure and also experiences were examined. This suggests that social acceptability of gender based role allocation and male dominant societal structure will lead to inequitable system where girls/women are likely to face discriminatory practices. This may impact on access to resources such as education, health services, financial support, and lifelong opportunities, including impacts on individual health and wellbeing. However, if interventions aimed at adolescents are introduced at appropriate times especially involving boys, then it may deliver substantial benefits for all sectors of the community. Similar findings are published from other countries. Research by De Meyer *et al*., (2014) involved young adolescents from Bolivia and Ecuador (N= 5,913) to investigate attitudes towards gender equality, sexual behaviour, experiences, and communication. Findings showed several benefits of gender equality such as use of contraception and positive experiences of intimate relations among sexually active adolescents. Importantly, those who were not sexually active also reported positive impacts on communication with their counterparts where sexual relations were deemed less necessary to maintain relationships. Findings were consistent in both boys and girls. Similar findings were recorded from an intervention study conducted in Brazil, where a link was observed between gender equitable norms and higher contraception use during recent sexual activity ( Pulerwitz *et al*., 2010). These wider benefits may help improve adolescent and women’s health as well as sexual and reproductive health outcomes.

Current evidence highlights the importance of developing effective interventions to address issues where adolescents will need to be involved from an early age ( Landry *et al*., 2020, Verma *et al*., 2006). These research findings including ours especially from male adolescents also indicate a worrying trajectory for their future behaviour towards women – there is substantial evidence that attitudes and behaviour developed in adolescent are carried into adulthood ( Blum *et al*., 2017). This enables these young men to influence a new cohort of adolescents and the cycle begins again ( Das *et al*., 2014). Studies elsewhere have reported some impact through educational programmes with young men ( Syed 2017, Jejeebhoy *et al*., 2017); however, literature also suggests that education is not enough, and thus interventions need to go further and be supported by more structural and legal interventions ( Jewkes *et al*., 2015). Nevertheless, it is important that work within rural communities addresses this issue and recognises that education approach is one of the key parts of interventions to improve gender attitudes of young males ( Casey *et al*., 2017, Jejeebhoy and Santhya 2018, Vyas *et al*., 2020). Our study also showed positive attitudes towards gender held by female adolescents – not unexpected – but highlights that gender equality work needs to recognise the importance of empowering young women to express their views about inequalities safely as well as effectively ( Santhya *et al*., 2014). It is also imperative that future work includes parents, especially mothers of young girls and fathers of young sons ( Dhar *et al*., 2019, Schroeder *et al*., 2019), as they can also have a positive effect on gender equality from an early age ( Basu *et al*., 2017, Kågesten *et al*., 2016).

Our gender equality scores were significantly lower in adolescents involved in some type of employment/paid work, suggesting the need for further development work that combines gender education and welfare programmes for poor and marginalised families to reduce discrimination and improve equality at family and community level. This links to the literature that finds an association between poor gender attitudes and lower educational attainment and development in male adolescents ( Gök *et al*., 2019) and emphasises the importance of early school interventions around gender equality to address such several co-exiting challenges ( Chae *et al*., 2020). It also means that communities themselves have a key role in helping these young boys and men develop more equitable beliefs towards gender, work that is ongoing in India and beyond, and it is showing some positive results ( Freudberg *et al*., 2018). Furthermore, differences seen in our rural population versus studies in urban settings reinforce the need to prioritise work in rural communities by government authorities and NGOs considering gender attitudes and beliefs of young men tend to be worse in poorer rural environments than urban areas ( Carter *et al*., 2016), and our data reinforces this pattern.

Our findings also demonstrate inequitable access to mobile phones in our study population where the majority of the boys owned a personal mobile phone (428 participants had a personal mobile of which 333 [77%] were boys). This is one of the examples of inequitable distribution of finances, resource allocations and preferences at family and community level suggesting parental preference to boys to offer such high value item, and decision-making power often centred around men and for men. We understand that our results are important considering the fifth SDG (Gender Equality) and provides useful research findings for a range of agencies working in India to accelerate progress towards SDGs. Such development work involving adolescents is also likely to generate long lasting impacts on existing and future generations to break the cycle of violence against girls/women and work towards establishing equitable and balanced societies.

### Strengths and limitations

Our key strengths include an ability to collect these data from rural areas resulting in a high response rate. To our knowledge this is the first survey from rural areas of the Maharashtra state involving adolescents’ boys and girls, which will help inform future research and development work in the state and elsewhere in India. Secondly, the study permitted development and testing of a survey instrument in a rural environment and provided a sufficiently reliable GE scale for future research such as intervention development and its evaluation. Lastly, as part of our wider project goal, we contributed to the development and capacity building of local human resource in low resource settings. Periodic training and mentoring sessions for data collection teams, staff, and collaborations with all project partners helped strengthen research capacities. However, our study has several limitations. Firstly, we do not exclude a possibility of response bias as all data were self-reported and participants were self-selected. It is possible that predominantly those adolescents who felt strongly about gender equality chose to participate in the study following reading our research/survey invitation.

Secondly, our findings are limited to the sub-group of adolescents aged 16 to 19 years. Given that independent informed decision-making ability was necessary to participate in the study, we were unable to enrol adolescents aged 10 to 15 years mainly due to ethical constraints. Survey results involving urban adolescents are likely to vary from our findings considering access to education, training and overall life opportunities are more for both urban boys and girls, thus our findings represent mainly rural populations. Lastly, we were unable to investigate possible effects of non-response bias on the generalisability of the survey results to the study population, because statistical information on the number and socio-demographic characteristics of adolescents aged 16 to 19 years old in the given study area was not available. Extrapolating demographic structure from the Census of India 2001 to the population of 68,000 in the given study area, we estimate that the study population of adolescents aged 16 to 19 is about 5304. Based on this estimation, the respondents represented 27% of the study population. Thus, we cannot exclude a possibility of non-response bias.

## Conclusion

Gender inequality is a major issue worldwide. Societal and cultural gender norms impact and shape individual attitudes and behaviours towards girl/women as well as expectations about their role and place in our society. Oppressive gender norms are often reflected in the social status of women and are often associated with violence against women. While oppressive gender norms are widespread globally, their impact on women’s health, development, and well-being is much more noticeable in patriarchal societies such as India. Any efforts to promote gender equality can hardly be effective without the involvement of men and boys from adolescent stages. This study provided important insights into the gender equality related knowledge, attitudes, and behaviours among boys and girls. Our findings suggest a need for community wide education and awareness programmes to promote gender equality especially from adolescence. It is important to note that the majority of the published literature on gender inequality and domestic violence focuses on adults (men and women), who are in intimate relationships. This warrants further research involving young people/adolescents to inform intervention work. Continuous improvements in measurement tools/scale are essential to the process of developing and evaluating interventions to accelerate progress towards gender equality.

## Data availability

### Underlying data

Data used in this study are confidential and are not available in the public domain. Ethics approvals provided permissions to use the dataset by specific individuals of the research team for stated purposes. Data from this project may be used further for future research by researchers/organisations involved in this work to conduct additional analyses.

If you wish to access this data, then please contact the corresponding author by email with the following information: a copy of your CV, research purpose/purpose for requesting data, and additional documents to support your request. Once these are reviewed by the researchers concerned, then you will receive a preliminary decision, and you will then be required to seek ethical approval from an ethical review board.

### Extended data

Figshare: ADolescents GEnder SurVey, REsponsible CoupLes EvaluatiOn, and Capacity Building Project in India (DEVELOP): A study protocol, https://dx.doi.org/10.6084/m9.figshare.8256050.v1 ( Ahankari *et al*., 2019).

This project contains the following extended data:

• Supplementary Files 1 to 5.pdf
• DEVELOP_Survey questionnaire Marathi.pdf
• DEVELOP_FGD Guide Marathi.pdf

Data are available under the terms of the Creative Commons Zero “No rights reserved” data waiver (CC0 1.0 Public domain dedication).
