# Supplementary material for: Knowledge, attitudes, and behaviours about gender equality: a cross-sectional survey of adolescents from rural India
Source: F1000Res. 2022 Sep 13;11:1045. [Version 1] doi: 10.12688/f1000research.124577.1 (PMC11393537; doi:10.12688/f1000research.124577.1)
Supplement: Peer review history

# Reviewer report: Reviewer response for version 1

**DOI:** 10.5256/f1000research.136781.r191000

**Published:** 2024-09-12

**Authors:** Showkeen Bilal Ahmad Gul

**Affiliations:** 1 University of Kashmir, Srinagar, Jammu and Kashmir, India

**Title:** The title of the research paper is clear and informative. It highlights the main subject of the study and provides essential information about the population and location of the research.

**Abstract:** The abstract follows a standard structure, including background, methods, results, and conclusions. It gives a concise overview of the study, enabling readers to understand the main purpose and findings quickly.

**Introduction:** The introduction provides a comprehensive overview of the importance of gender equality in achieving Sustainable Development Goals (SDGs) and its relevance to India's context. It highlights the challenges faced by women and girls in India due to gender-based discrimination and inequalities. The introduction starts with a strong opening by mentioning the importance of gender equality as the fifth Sustainable Development Goal (SDG) and its linkage to other SDGs. This hook immediately engages the reader's interest and highlights the broader significance of the research.

 Use of References: The introduction makes good use of references to support the points being made. However, some of the references are relatively old (e.g., 2011, 2012). It would be beneficial to include more recent references to provide up-to-date context and data on gender inequality in India.

 Clarity in Problem Statement: The introduction effectively communicates the problem statement - the prevalence of gender-based discrimination and inequalities in India, especially in rural areas. The use of statistics, such as India's rank in the gender inequality index, adds credibility to the research.

 Mention of Research Gap: The introduction successfully identifies the research gap - the lack of published data on gender equality-related knowledge, attitudes, and behaviors among adolescents in rural areas of Maharashtra, India. This helps establish the significance of the study and its contribution to the existing literature.

 Detailed Description of Study Setting: The introduction gives a detailed description of the study setting, including the selection of villages in the Osmanabad district of Maharashtra, India. However, it might be useful to mention briefly why this particular region was chosen and its relevance to the research objectives.

**Methodology:** Clarity of Adaptation Process: The methodology provides a clear description of how the survey instrument was adapted from the Gender Equitable Measurement (GEM) Scale used in urban settings to fit the rural population. However, further elaboration on the specific reasons for excluding and adding certain items could enhance the transparency of the adaptation process.

 Inclusion of Stakeholders: Involving project partners, research team members, project staff, and local stakeholders in adapting the survey instrument is commendable. However, the roles and contributions of these stakeholders could be better outlined to demonstrate their significance in the process.

 Face Validity: Discussing the specific themes and feedback received from the over fifty adolescents during the face validity check would strengthen the credibility of the instrument's content validity.

 Translation and Cultural Adaptation: While the translation of the survey instrument into Marathi language is mentioned, it would be beneficial to detail any challenges encountered during this process and how they were addressed to ensure linguistic and cultural equivalence.

 Data Collection Process: The data collection process is well-detailed, outlining the roles of the data collection teams, the senior research coordinator, and the project manager. However, it would be beneficial to clarify how the initial list of eligible participants was determined and the criteria used to select the 139 questionnaires for removal during quality checks.

 Informed Consent: The informed verbal consent process is adequately described. To enhance transparency, consider adding a brief statement on how participants were assured of their right to decline participation without any consequences.

 Data Collection Locations: While you mention that data collection sites varied, more information about how these sites were selected and whether they represented diverse demographics within the rural areas would provide context to the reader.

 Demographics of Study Participants: The description of the study participants' demographics provides a clear overview. However, further elaboration on how the characteristics of the participants align with the rural Indian setting and any potential implications of these demographics on the study's findings could enhance the discussion.

 Gender Differences: The finding of statistically significant gender differences in GE related knowledge, attitudes, and behaviors is well-discussed. To strengthen this discussion, you could briefly reflect on possible underlying societal factors that might contribute to these differences.

 Comparative Studies: The comparison of your study's results with findings from other studies, particularly within India, is valuable. You could further expand on how these findings collectively contribute to a broader understanding of gender attitudes and behaviors among adolescents in various cultural contexts.

 Importance of Early Interventions: The emphasis on the importance of early interventions to address gender attitudes and behaviors is well-articulated. However, you could elaborate on specific strategies that have been proven effective in changing these attitudes and behaviors among adolescents, potentially citing examples from successful intervention programs.

 Implications for Future Interventions: The discussion of implications for future interventions, especially involving parents and communities, is noteworthy. Consider providing a concise list of recommendations for policymakers, NGOs, and other stakeholders based on your findings.

 Limitations: The discussion of limitations is thorough and transparent. To further enhance this section, you could briefly touch on any potential implications of the identified limitations on the generalizability of your findings to other rural Indian settings or different cultural contexts.

Is the work clearly and accurately presented and does it cite the current literature?

Yes

If applicable, is the statistical analysis and its interpretation appropriate?

Partly

Are all the source data underlying the results available to ensure full reproducibility?

Yes

Is the study design appropriate and is the work technically sound?

Yes

Are the conclusions drawn adequately supported by the results?

Yes

Are sufficient details of methods and analysis provided to allow replication by others?

Partly

Reviewer Expertise:

Gender equality and equity, pre-primar Education, elementary education, women and violence

I confirm that I have read this submission and believe that I have an appropriate level of expertise to confirm that it is of an acceptable scientific standard.

# Reviewer report: Reviewer response for version 1

**DOI:** 10.5256/f1000research.136781.r249850

**Published:** 2024-03-21

**Authors:** Natalia López Hornickel

**Affiliations:** 1 Department of Education, University of Bath, Bath, England, UK

This is an interesting contribution of analysis of gender attitudes in a rural area in India. It is critical to know more than just urban sectors in countries. Which makes it a good contribution. 

 The data presented in this study is novel, offering fresh insights into the topic. It's worth noting that this data was collected as part of a previous project, which provides a valuable context for its interpretation. 

 I have some comments that I believe could be useful for you, the authors, to further improve the article. 

 In the introduction, the gender unequal situation in India is well described, considering systemic discrimination and gender violence. Nevertheless, it is never mentioned why it is important to study attitudes. Why can understanding attitudes contribute to analysing this situation? This is more explained in the discussion section, but I think it is important to establish this at the beginning of the text. 

 Regarding the data, the process of creating the instrument is well explained. Nevertheless, there is little information about the sampling. Was it a random selection of the participants? Was it stratified? This is critical if the results want to be expanded to the community under study. Otherwise, they are not representative. 

 Regarding the analysis, I don't understand why the authors decided to put descriptives in the same table as the regression results. I would separate both to be more clear. I would also put Table 2 as Table 1 and then the regression analysis.     

 The table containing the regression should describe the outcome under study.

 - I missed an explanation of their inclusion in the predictors included in the analysis of regression. Why are these predictors important for the context under study?

 This could enrich the discussion as well.

Is the work clearly and accurately presented and does it cite the current literature?

Yes

If applicable, is the statistical analysis and its interpretation appropriate?

Partly

Are all the source data underlying the results available to ensure full reproducibility?

Partly

Is the study design appropriate and is the work technically sound?

Yes

Are the conclusions drawn adequately supported by the results?

Yes

Are sufficient details of methods and analysis provided to allow replication by others?

Yes

Reviewer Expertise:

Gender attitudes, citizenship studies, large scale assessments.

I confirm that I have read this submission and believe that I have an appropriate level of expertise to confirm that it is of an acceptable scientific standard, however I have significant reservations, as outlined above.

# Reviewer report: Reviewer response for version 1

**DOI:** 10.5256/f1000research.136781.r210374

**Published:** 2023-10-31

**Authors:** Nitasha Nagaraj

**Affiliations:** 1 Department of Prevention and Community Health, The George Washington University, Washington, District of Columbia, USA

This manuscript is written very well and thought out. A few places that I believe need further clarification include:

1. Table 1 should be split up. It would be more clear if it only consisted of the sample characteristics and then the logistic models be moved to a separate table. 
2. In the analysis, the authors indicate a low, moderate, and high score. While there is assumption that a high score would mean higher gender equitable KAB, this is not mentioned anywhere in the paper. This should be clarified.
3. Table 2 includes frequencies of all the statements. Were there any gender differences? age differences? It would be good to see these in a table.
4. There is also reference to mean scores - this information should be in a table format and if there were differences in each of these categories based on any of the socio-demographics. 

Is the work clearly and accurately presented and does it cite the current literature?

Yes

If applicable, is the statistical analysis and its interpretation appropriate?

Partly

Are all the source data underlying the results available to ensure full reproducibility?

Yes

Is the study design appropriate and is the work technically sound?

Yes

Are the conclusions drawn adequately supported by the results?

Yes

Are sufficient details of methods and analysis provided to allow replication by others?

Partly

Reviewer Expertise:

Evaluation, maternal and child health, adolescent health, voice and agency

I confirm that I have read this submission and believe that I have an appropriate level of expertise to confirm that it is of an acceptable scientific standard, however I have significant reservations, as outlined above.
